# Overactive bladder medications and risk of emergency hospital admissions with delirium in adults without dementia: self-controlled case series

**DOI:** 10.1093/ageing/afaf308

**Published:** 2025-10-27

**Authors:** Kathryn Richardson, Irene Petersen, Katharina Mattishent, Oby Otu Enwo, Nick Steel, Duncan Edwards, Jalesh N Panicker, Stuart Irving, Christopher Fox, Louise Robinson, Yoon Kong Loke

**Affiliations:** Norwich Medical School, University of East Anglia, Norwich, UK; Department of Primary Care and Population Health, University College London, London, UK; Norwich Medical School, University of East Anglia, Norwich, UK; Norwich Medical School, University of East Anglia, Norwich, UK; Norwich Medical School, University of East Anglia, Norwich, UK; Department of Public Health and Primary Care, University of Cambridge, Cambridge, UK; Department of Uro-Neurology, The National Hospital for Neurology and Neurosurgery, University College London NHS Foundation Trust, London, UK; Department of Translational Neuroscience and Stroke, UCL Queen Square Institute of Neurology, Faculty of Brain Sciences, University College London, London, UK; Norwich Medical School, University of East Anglia, Norwich, UK; University of Exeter Medical School, University of Exeter, Exeter, UK; Population Health Sciences Institute, Newcastle University, Newcastle upon Tyne, UK; Norwich Medical School, University of East Anglia, Norwich, UK

**Keywords:** dementia, pharmacoepidemiology, anticholinergic, medication use, antimuscarinic, overactive bladder, self-controlled case series, older people

## Abstract

**Aims:**

We examined whether anticholinergic overactive bladder (OAB) medications are associated with emergency hospital admissions with delirium in adults without dementia.

**Setting:**

England’s primary care linked to inpatient records.

**Methods:**

The source population comprised 215 293 adults initiating anticholinergic OAB medications (e.g. oxybutynin, solifenacin and tolterodine) during July 2010–December 2019 when aged ≥50 years, without dementia, severe mental illness or <12 months registration. We conducted self-controlled case-series including 1831 men and 1954 women with emergency hospital admissions with delirium. Incidence rate ratios (IRR) were estimated in risk periods during 6 months before and 12 months after initiating OAB medications, adjusted for time-varying age, separately in men and women.

**Results:**

The risk of delirium admissions was elevated for the first 1–30 days of prescriptions [IRR 1.54 (95% CI 1.30–1.82) for men, 1.44 (1.22–1.70) for women] and whilst reducing over time for women [1.10 (0.94–1.29) for 91–365 days of prescriptions], it remained elevated for men [1.38 (1.17–1.64)]. There was some evidence of greater delirium IRRs in older men and men initiating higher dosages. In secondary analysis of 502 adults initiating mirabegron (non-anticholinergic beta-3 receptor agonist), the delirium IRRs during the first 1–30 and 31–90 days of prescriptions were 1.15 (0.76–1.75) and 0.72 (0.47–1.09).

**Conclusion:**

We observed increased hospital admission rates with delirium for adults without dementia whilst prescribed OAB anticholinergics, but not whilst prescribed mirabegron. Delirium risk remained raised for longer for men and was greater in older men. Alternative management options for OAB in older people should be considered before prescribing anticholinergic medications.

## Key Points

- Anticholinergic bladder drugs are linked to increased hospital admissions with delirium in adults without dementia.
- Raised admission rates with delirium persist throughout the anticholinergic treatment period in men, but not women.
- In men, there is some evidence of stronger associations with older age and higher initial dosage.
- Elevated hospital admission rates with delirium are similar across common anticholinergic bladder drugs.
- There was no evidence for an association between mirabegron prescribing and hospital admissions with delirium.

## Introduction

Delirium is common in older hospitalised patients, affecting around 23% of inpatients [1]. It is characterised by an acute onset of disturbed cognition, consciousness and perception. Delirium can manifest in agitation and confusion (hyperactive delirium), withdrawal and drowsiness (hypoactive delirium) or both [2]. Delirium is associated with longer inpatient stays, increased dementia incidence, mortality and hospital-associated complications [2].

The pathophysiology of delirium is multifactorial and not fully understood, but leading hypotheses focus on neuroinflammation, chronic stress and neurotransmitter imbalance [3, 4]. Of the neurotransmitters, the key hypothesised mechanism for delirium is central cholinergic deficiency [5–7]. Many commonly prescribed medications have anticholinergic properties and studies suggest greater anticholinergic burden is associated with increased risks of cognitive impairment and dementia [8, 9].

Overactive bladder (OAB) syndrome is characterised by urinary urgency, frequency and nocturia and with or without incontinence. OAB symptoms are common in the older population, affecting around 17% of the over 40s [10], and significantly impact quality of life [11]. OAB is caused by overactivity of the detrusor [12], which is controlled by the parasympathetic nervous system through cholinergic muscarinic receptors. As such, anticholinergic drugs blocking muscarinic receptors (e.g. oxybutynin, solifenacin and tolterodine) are part of the therapeutic armamentarium for OAB [13].

However, age-related changes in the blood–brain barrier may allow anticholinergics to cause central nervous system effects [13, 14], ranging from headache to cognitive impairment and psychosis [8, 9, 13, 15]. Prescribers and patients should be fully informed regarding potential adverse cognitive effects when weighing up initiating bladder medications. Equally, healthcare professionals need guidance to identify precipitating factors when assessing older people presenting with delirium.

In addition to anticholinergic medications, mirabegron (a selective beta-3 adrenoceptor agonist without anticholinergic properties) was licensed for OAB in the UK in 2012. A 12-week placebo-controlled trial did not demonstrate any cognitive adverse effects [16]. Increased delirium rates were observed with oxybutynin versus mirabegron in a small Ontario database study [17], although not reaching statistical significance, this requires further evaluation. Some studies find the total anticholinergic burden of medications is, in general, associated with greater delirium risk [18], but most studies are underpowered or limited by residual confounding between patients.

To better understand one mechanism whereby OAB medication exposure may increase long-term cognitive decline in people without dementia, we examined whether OAB medications increase the risk of acute cognitive adverse effects (namely, delirium) in this population.

## Methods

### Study design and setting

We conducted self-controlled case series (SCCS) analyses to compare the incidence of delirium on emergency hospital admission when prescribed anticholinergic OAB medications, to reduce the impact of confounding between patients. A background to SCCS approaches is described in Supplementary Appendix 1. We used Clinical Practice Research Database Aurum (CPRD) linked to Hospital Episode Statistics Admitted Patient Care (HES) and Index of Multiple Deprivation (IMD) datasets. CPRD containing anonymised registration, prescription, diagnosis and referral data from primary care, including 41.2 million patient records and is representative of England’s population [19]. HES includes information on demographics, diagnoses [coded using International Classification of Diseases 10th revision (ICD-10)] and procedures undertaken during hospital admissions [20]. IMD combines indicators of housing, employment, income, education and environment at the patient’s postal code level [21].

### Participants

We identified individuals aged ≥50 years with a first anticholinergic OAB prescription (oxybutynin, solifenacin, tolterodine, darifenacin, fesoterodine, propiverine and trospium) between July 1 2010 and 31 December 2019 in England. At first prescription, we excluded patients with history of dementia, severe mental illness, antipsychotic (excluding prochlorperazine) prescription in the last year or <12 months registration in CPRD.

### Outcome

Our outcome was emergency admission to hospital with delirium; we did not evaluate delirium acquired during hospital stays as inpatient prescribing data were unavailable through CPRD. HES diagnoses are recorded by calendar date only and not time of day. To allow for delays in coding we included delirium recorded on admission date or the following day. However to exclude delirium post surgery, we excluded delirium cases on the day after admission if there had been surgery by that day. We started recording admissions in 2010 when guidelines were introduced to improve delirium recording [22], and ended before 2020 to exclude delirium due to COVID-19. We defined delirium using ICD-10 codes F05 or R41.0. To ensure independent events, we only included the first eligible delirium admission within the SCCS study period [23].

### Exposures

Our approach to prepare OAB prescription data and estimate treatment episodes is described in Supplementary Appendix 2. We considered a new prescription represented a new treatment episode if it occurred >60 days after the previous prescription end date. Episode end was defined as the last prescription end date plus a 30-day grace period.

Daily dosage was estimated as prescription dose × dose frequency per day/defined daily dose (DDD). We used World Health Organisation DDD values per drug, which are the assumed average daily maintenance dose based on its main indication in adults. The British National Formulary recommends similar daily doses for adults with OAB, but to initially halve doses for older patients [24]. We coded first prescriptions into half-adult dosages (≤0.5 DDDs), adult dosages (>0.5–1 DDD) or greater than adult dosages (>1 DDD).

### Covariates

SCCS designs implicitly control for time-invariant confounders [23]. We adjusted for time-varying age in 40 gender-specific bands (defined by quantiles of delirium admission age). In secondary analyses, we adjusted for time-varying episodes of urinary tract infection (UTI), defining new episodes after >30 days between CPRD UTI records, and assumed episodes continued for 14 days beyond their last record.

### Statistical analysis

The SCCS study period included only 6 months before and ≤12 months after the first OAB prescription, to reduce the impact of time-varying confounding in this older population (Supplementary eFigure 1). We censored patients at the first of: 12 months after first prescription, death, 31 December 2019, leaving the General Practitioner (GP) practice and last GP data collection date.

We examined the exposure period in risk windows: 1–30, 31–90 and 91–365 days after the episode first prescription date. All other observation times constituted the baseline (unexposed) period, except for a 30-day pre-exposure and 60-day post-exposure period. The pre-exposure window addresses potential bias from delirium conditioning the probability of OAB prescribing [23]. The 60 post-exposure washout period allows for uncertainty in when patients stop taking medications.

We found evidence that delirium censored the observation period, so applied the SCCS extension for event-dependent observation periods to estimate incidence rate ratios (IRRs) adjusted for time-varying age [25]. Age and dementia diagnosis after delirium admission were designated as covariates predictive of censoring. We prespecified reporting findings separately by men and women, due to gender differences in reasons for urinary issues, possible differential risks of delirium and cognitive impairment, and vulnerability to anticholinergic effects [26–28].

As secondary analysis, we also adjusted for time-varying UTI, including 30-day pre-exposure periods (representing symptomatic UTI). We stratified analyses exploring whether delirium risk varied by age, initial drug and dosage. Likelihood ratio tests were used to compare models with and without interaction terms [29]. We report IRRs estimated using model interaction terms.

### Secondary analysis—mirabegron and delirium

Modifications to the study design above for patients initiating mirabegron are described in Supplementary Appendix 3 and Supplementary eFigure 2.

### Sensitivity analyses

We compared our findings to those using standard SCCS models, and applied common recommendations when events increase the censoring probability [23], namely that of excluding patients who (i) died or (ii) died or left the practice within 90 days after delirium. We performed sensitivity analyses with (i) an additional 30-day pre-exposure period, (ii) without the 30-day grace period for treatment duration end and (iii) excluding those diagnosed with dementia during their delirium admission. For the mirabegron analysis, we additionally excluded those with any history of OAB anticholinergic prescriptions.

Data management was performed using Stata version 18.0. The SCCS package version 1.7 in RStudio and R version 4.0.4 was used for SCCS analyses.

## Results

Of 215 293 eligible patients initiating anticholinergic OAB medications, 1831 men and 1954 women were admitted with delirium during the SCCS period (Supplementary eFigure 1). Of 17 009 eligible patients initiating mirabegron, 245 men and 257 women were admitted with delirium. The mean (SD) ages of patients initiating anticholinergics and mirabegron included in analyses were 81.1 (9.3) and 81.9 (8.8) years (Table 1). Neurological conditions were common, and more cardiovascular diseases observed in men and more UTIs and concurrent antidepressant use in women (Table 1). Frequent primary reasons for hospital admission besides delirium, were UTI, other infections, fractures and falls and injuries (Supplementary eTable 1).

**Table 1:** Characteristics of patients prescribed either anticholinergic overactive bladder drugs or mirabegron and admitted with delirium. Values are numbers and percentages unless stated otherwise.

Characteristics at first overactive bladder prescription	Initiating an OAB anticholinergic				Initiating mirabegron			
	Men (*N* = 1831)		Women (*N* = 1954)		Men (*N* = 245)		Women (*N* = 257)	
	*n*	%	*n*	%	*n*	%	*n*	%
Age, years^a^	80.4	9.0	81.8	9.6	81.6	8.7	82.2	8.9
White ethnicity^b^	1720	93.9%	1869	95.6%	226	92.2%	249	96.9%
Index of multiple deprivation decile^a^	5.5	5.2	5.4	4.1	5.1	2.9	5.5	6.5
Stroke/transient ischaemic attack	569	31.1%	573	29.3%	90	36.7%	65	25.3%
Parkinson’s disease	158	8.6%	78	4.0%	28	11.4%	28	10.9%
Epilepsy	85	4.6%	95	4.9%	14	5.7%	11	4.3%
Other neurological condition^c^	44	2.4%	62	3.2%	13	5.3%	14	5.4%
Diabetes	622	34.0%	537	27.5%	87	35.5%	85	33.1%
Coronary heart disease	1087	59.4%	953	48.8%	140	57.1%	124	48.2%
UTI (last 6 months)	577	31.5%	843	43.1%	71	29.0%	116	45.1%
**Prescriptions in last 90 days**								
Tricyclic antidepressant	159	8.7%	265	13.6%	7	2.9%	29	11.3%
Selective serotonin reuptake inhibitor	330	18.0%	449	23.0%	30	12.2%	37	14.4%
Opioid	633	34.6%	826	42.3%	46	18.8%	80	31.1%
Lipid regulating medication	1006	54.9%	933	47.7%	98	40.0%	76	29.6%
Antiplatelet	776	42.4%	731	37.4%	58	23.7%	50	19.5%
Antibiotic	820	44.8%	958	49.0%	60	24.5%	84	32.7%
Alpha blocker (any history)	1157	63.2%	N/A		67	27.3%	N/A	

^a^Mean (SD).

^b^Missing data on ethnicity for 11 patients.

^c^Any history of other neurological conditions (multiple sclerosis, motor neuron disease, multiple system atrophy, hemiplegia, hydrocephalus, brain injury, brain tumour) in patients without history of stroke, transient ischemic attack (TIA), Parkinson’s disease or epilepsy.

### Anticholinergic overactive bladder medications and delirium

Of the 3785 delirium admissions, 1722 occurred whilst prescribed OAB anticholinergics, and 356 during the first 30-days of treatment (Supplementary eTable 2). During the entire follow-up to 31 December 2019, 14 250 emergency admissions with delirium were recorded between treatment initiation for patients without dementia (Supplementary eTable 3).

Delirium rates increased during the first 1–30 days of treatment [IRR 1.54 (95% CI 1.30–1.82) for men, 1.44 (1.22–1.70) for women] and whilst fading over time for women, they remained for men [1.38 (1.17–1.64) for 91–365 days after initiation] (Table 2). Delirium IRRs varied significantly by gender (*P* = .005 likelihood ratio test). The delirium IRRs for men remain elevated for the entire drug exposure period when broken down into five exposure periods (Supplementary eTable 4). The delirium IRRs were attenuated, but not eliminated, after adjustment for time-varying UTI (Table 2). For example, during the first 1–30 days of prescriptions, the IRR (95% CI) was 1.29 (1.08–1.55) for men and 1.23 (1.03–1.47) for women.

**Table 2:** IRRs for emergency hospital admission with delirium for patients initiating anticholinergic overactive bladder medications, by gender.

Time period	Men				Women			
	Delirium	Patient	IRR (95% CI)		Delirium	Patient	IRR (95% CI)	
	Events	years	Adjusted for age	Adjusted for age & UTI	Events	years	Adjusted for age	Adjusted for age & UTI
Baseline	723	1113.2	1.00	1.00	829	1239.4	1.00	1.00
Pre-exposure: 30 days before	125	168.3	1.16 (0.96–1.41)	1.13 (0.93–1.39)	83	180.5	0.69 (0.55–0.87)	0.69 (0.54–0.87)
Drug exposure: 1–30 days	175	165.6	1.53 (1.29–1.82)	1.29 (1.08–1.55)	182	178.0	1.43 (1.22–1.69)	1.23 (1.03–1.47)
Drug exposure: 31–90 days	248	256.5	1.37 (1.17–1.60)	1.24 (1.05–1.46)	257	277.9	1.28 (1.10–1.49)	1.24 (1.06–1.45)
Drug exposure: 91–365 days	422	440.2	1.38 (1.17–1.64)	1.25 (1.05–1.50)	438	510.9	1.10 (0.94–1.29)	1.03 (0.87–1.22)
Post-exposure: first 60 days	138	173.1	0.97 (0.80–1.18)	0.86 (0.70–1.05)	165	191.4	1.07 (0.90–1.28)	1.02 (0.85–1.23)

#### Delirium risk by anticholinergic drug, dosage and age

Of the 4246 treatment episodes, 3903 (92%) began with oxybutynin, solifenacin or tolterodine prescriptions, and 2778 (71%) remained prescribed only that drug. The mean (SD) initial dosage of oxybutynin, solifenacin and tolterodine was 0.41 (0.25), 1.09 (0.35) and 0.82 (0.30) DDDs per day. There were no significant differences in delirium IRRs by drug in men (*P* = .66 for likelihood ratio test) or women (*P* = .16), but this may partly reflect dosage variation (Supplementary eTable 5).

Delirium IRRs in the first 30 days were greater for adult doses compared to half-adult doses in men (Table 3), albeit not reaching statistical significance (*P* = .09 for men and *P* = .29 for women). The delirium IRR (95% CI) for the first 30 days in men prescribed half-adult doses or adult doses compared to baseline, were 1.27 (0.96–1.69) and 1.73 (1.39–2.14). Overall, however, there were no statistically significant differences in delirium IRRs by initial DDD category in men (likelihood ratio test *P* = .41) or women (*P* = .19).

**Table 3:** IRRs for emergency hospital admission with delirium for patients initiating anticholinergic overactive bladder medications, by gender, initial dosage.^a^

Time period	Delirium	IRR (95% CI)		Delirium	IRR (95% CI)	
	Events	Age adjusted	Age & UTI adjusted	Events	Age adjusted	Age & UTI adjusted
Men	Initial dosage ≤ 0.5 DDDs			Initial dosage > 0.5 DDDs		
Baseline	310	1.00	1.00	413	1.00	1.00
Pre-exposure: 30 days before	48	1.07 (0.78–1.46)	0.92 (0.67–1.27)	77	1.23 (0.96–1.58)	1.03 (0.80–1.34)
Drug exposure: 1–30 days	60	1.27 (0.96–1.69)	1.11 (0.82–1.50)	115	1.73 (1.39–2.14)	1.48 (1.18–1.86)
Drug exposure: 31–90 days	101	1.39 (1.09–1.77)	1.28 (1.00–1.65)	147	1.36 (1.11–1.66)	1.23 (0.99–1.52)
Drug exposure: 91–365 days	140	1.19 (0.91–1.56)	1.08 (0.82–1.43)	282	1.51 (1.23–1.86)	1.38 (1.11–1.72)
Post-exposure: first 60 days	58	0.90 (0.67–1.20)	0.81 (0.60–1.11)	80	1.02 (0.79–1.31)	0.90 (0.69–1.18)
Women	Initial dosage ≤ 0.5 DDDs			Initial dosage > 0.5 DDDs		
Baseline	367	1.00	1.00	462	1.00	1.00
Pre-exposure: 30 days before	31	0.60 (0.41–0.87)	0.50 (0.34–0.73)	52	0.76 (0.56–1.01)	0.67 (0.50–0.91)
Drug exposure: 1–30 days	70	1.29 (0.99–1.68)	1.13 (0.86–1.49)	112	1.55 (1.25–1.92)	1.37 (1.09–1.71)
Drug exposure: 31–90 days	116	1.37 (1.10–1.71)	1.32 (1.05–1.67)	141	1.23 (1.00–1.50)	1.19 (0.97–1.48)
Drug exposure: 91–365 days	191	1.30 (1.02–1.65)	1.22 (0.95–1.56)	247	0.98 (0.80–1.21)	0.93 (0.75–1.15)
Post-exposure: first 60 days	77	1.09 (0.85–1.41)	1.08 (0.83–1.41)	88	1.03 (0.82–1.31)	0.99 (0.77–1.28)

^a^The WHO DDDs for oxybutynin, solifenacin and tolterodine are 15 mg, 5 mg and 4 mg per day.

There was evidence of delirium rates varying by age (*P* = .005 likelihood ratio test for men, *P* = .04 for women). Delirium IRRs in the first 30 days were 1.16 (0.78–1.72), 1.55 (1.19–2.01) and 1.77 (1.34–2.34) for men aged 50–74, 75–84 and 85+ years at OAB medication initiation (*P* = .07 for trend) (Table 4).

**Table 4:** IRRs for emergency hospital admission with delirium for patients initiating anticholinergic overactive bladder medications, by gender and age group.

Time period and subgroup	Delirium	IRR (95% CI)		Delirium	IRR (95% CI)		Delirium	IRR (95% CI)		*P* for trend^a^	
	Events	Age adjusted	Age & UTI adjusted	Events	Age adjusted	Age & UTI adjusted	Events	Age adjusted	Age & UTI adjusted	Age adjusted	Age & UTI adjusted
Men	Age 50–74 years			Age 75–84 years			Age 85+ years				
Baseline	167	1.00	1.00	306	1.00	1.00	250	1.00	1.00		
Pre-exposure: 30 days before	31	1.24 (0.84–1.84)	1.10 (0.73–1.66)	55	1.20 (0.89–1.61)	1.03 (0.76–1.41)	39	1.06 (0.75–1.50)	0.85 (0.60–1.22)	.35	.34
Drug exposure: 1–30 days	31	1.16 (0.78–1.72)	0.91 (0.60–1.39)	76	1.55 (1.19–2.01)	1.40 (1.07–1.85)	68	1.77 (1.34–2.34)	1.53 (1.14–2.06)	.07	.04
Drug exposure: 31–90 days	59	1.34 (0.97–1.83)	1.19 (0.85–1.66)	82	1.02 (0.78–1.32)	0.95 (0.72–1.26)	107	1.87 (1.46–2.39)	1.67 (1.29–2.17)	.05	.05
Drug exposure: 91–365 days	104	1.24 (0.89–1.71)	1.04 (0.74–1.47)	170	1.26 (0.96–1.64)	1.19 (0.89–1.57)	148	1.67 (1.25–2.23)	1.57 (1.15–2.12)	.29	.13
Post-exposure: first 60 days	31	1.02 (0.69–1.53)	0.86 (0.56–1.32)	58	0.90 (0.67–1.21)	0.86 (0.63–1.17)	49	1.00 (0.73–1.38)	0.88 (0.62–1.23)	.72	.61
Women	Age 50–74 years			Age 75–84 years			Age 85+ years				
Baseline	150	1.00	1.00	314	1.00	1.00	365	1.00	1.00		
Pre-exposure: 30 days before	14	0.63 (0.36–1.10)	0.53 (0.30–0.94)	31	0.69 (0.47–1.00)	0.59 (0.40–0.86)	38	0.72 (0.51–1.01)	0.63 (0.44–0.90)	.53	.46
Drug exposure: 1–30 days	35	1.52 (1.04–2.23)	1.27 (0.85–1.90)	64	1.36 (1.03–1.80)	1.22 (0.91–1.64)	83	1.46 (1.14–1.88)	1.29 (0.99–1.68)	.43	.47
Drug exposure: 31–90 days	36	1.07 (0.73–1.57)	1.05 (0.71–1.55)	103	1.39 (1.10–1.77)	1.39 (1.08–1.79)	118	1.28 (1.02–1.60)	1.21 (0.95–1.53)	.86	.96
Drug exposure: 91–365 days	84	1.27 (0.88–1.81)	1.28 (0.88–1.85)	172	1.19 (0.91–1.54)	1.13 (0.86–1.49)	182	0.96 (0.75–1.23)	0.86 (0.66–1.11)	.18	.05
Post-exposure: first 60 days	41	1.50 (1.05–2.16)	1.66 (1.14–2.42)	48	0.83 (0.61–1.14)	0.82 (0.59–1.15)	76	1.07 (0.82–1.39)	0.96 (0.73–1.27)	.50	.14

^a^A linear trend with age was tested, by examining interactions with continuous age.

### Mirabegron and delirium risk

The 502 adults initiating mirabegron with a delirium admission were initially prescribed a mean (SD) of 0.74 (0.27) DDDs. The IRRs (95% CI) for delirium during the first 1–30 days of prescriptions were 1.15 (0.76–1.75) adjusted for age and 1.11 (0.72–1.73) also adjusted for time-varying UTIs (Table 5). There was no evidence of increased delirium rates during the following 31–545 days of prescriptions. There was no evidence of gender differences, either overall (*P* = .26 likelihood ratio test) or in the first 1–30 days of prescriptions (*P* = .80).

**Table 5:** IRRs for emergency hospital admission with delirium for patients initiating mirabegron.

			IRR (95% CI)	
Time period	Delirium events	Patient years	Adjusted for age and anticholinergic OAB exposure	Adjusted for age, anticholinergic OAB exposure & UTI
Baseline	266	598.0	1.00	1.00
Pre-exposure: 31–60 days before	34	41.2	1.80 (1.25–2.59)	1.61 (1.09–2.38)
Pre-exposure: 30 days before	11	41.2	0.54 (0.29–0.99)	0.44 (0.23–0.83)
Drug exposure: 1–30 days	25	40.8	1.15 (0.76–1.75)	1.11 (0.72–1.73)
Drug exposure: 31–90 days	26	66.9	0.72 (0.47–1.09)	0.71 (0.46–1.09)
Drug exposure: 91–545 days	110	192.5	0.87 (0.63–1.18)	0.81 (0.59–1.13)
Post-exposure: first 60 days	30	45.4	1.08 (0.73–1.61)	0.96 (0.63–1.47)

### Sensitivity analysis

The occurrence of delirium events led to a reduced subsequent observation period due to greater censoring from mortality and moving GP practice (Supplementary eFigure 4 and Supplementary eFigure 5). Delirium IRRs from standard SCCS models were generally greater than from SCCS extension models, but were influenced by excluding those censored within 90 days of the outcome due to death or moving GP (Supplementary eTable 6 and Supplementary eTable 7).

Delirium rates during anticholinergic exposed periods increased slightly when we extended the pre-exposure period (Supplementary eTable 8). Removing the 30-day prescription end grace period had minimal impact on the drug exposure IRRs. However, IRRs increased during the 60-day post-exposure period for women suggesting some continued taking their medications (Supplementary eTable 9). Associations were similar when excluding those diagnosed with dementia during their delirium admission (Supplementary eTable 10). The sensitivity analyses had little impact on mirabegron IRRs (Supplementary eTable 11 and Supplementary eTable 12).

## Discussion

In this SCCS of 4287 adults without dementia, we observed increased emergency hospital admissions with delirium whilst prescribed anticholinergic OAB medications. Risk ratios were higher in men, especially older men and men prescribed higher initial dosages. We did not detect statistical differences between anticholinergic drugs. Despite limited mirabegron exposure, little evidence suggested increased delirium risk with this medication.

### Strengths and weaknesses

The study strengths include a large real-world population, use of both primary and secondary care records and the SCCS design to reduce confounding. We accounted for time-varying age and UTI, although residual time-varying confounding remains possible. UTI was not adjusted for main analyses due to its potential mediating role [30]. Sensitivity analyses supported the robustness of findings. Delirium events reduced subsequent follow-up, but extended SCCS methods addressed this.

Limitations include probable under-reporting of delirium, despite improved UK recording since 2010 [22, 31–33]. Our delirium definition excluded encephalopathy terms consistent from formal delirium definitions [4, 34], but included non-specific confusion code ‘disorientation unspecified’ due to historic UK use [31]. Delirium admission incidence in those aged ≥65 years was 0.24% within 30 days of anticholinergic initiation, and 7.5% of emergency admissions before 2020 and dementia diagnosis had delirium noted. Estimates of a 30-day delirium incidence rate of 0.30% in Canada [17], and pooled 23% delirium rate in older inpatients [1], were higher, but probably influenced by including people with dementia. We explored including delirium noted in primary care records, but found it rarely recorded. Our delirium outcome is likely under-reported; hence some associations may be biased towards the null [35].

The prescribing records stem from primary care and exclude secondary care prescriptions. However, initial OAB treatment usually occurs in primary care and those initiated in secondary care are usually continued in primary care. Medication adherence is unknown, potentially underestimating our delirium IRRs. A 30-day grace period in prescription duration may cause minor exposure misclassification, but sensitivity analyses showed minimal impact. Missing dose frequency data were infrequent, addressed using sensible imputations and expected to have little effect on dosage category assignment. Subgroup analyses, especially for mirabegron, may be underpowered, and those results should be interpreted with caution. Dementia is underdiagnosed and diagnosis can be delayed [36], so some study participants may have dementia and potentially delirium accelerates that diagnosis. Although excluding those with dementia diagnosed during the delirium admission had little effect on our findings, many of the study participants may have some dementia pathology, and it still could be the case that the anticholinergic exposure unmasks the underlying pathology in some patients.

### Comparisons with other studies

A Canadian cohort study reported an odds ratio for delirium within 30-days initiating oxybutynin of 1.28 (0.84–1.96), but lacked statistical power with only 84 eligible subsequent delirium admissions (we detected 357 such delirium admissions) [17]. They reported a hazard ratio for delirium for continuous use of ‘newer OAB anticholinergics’ (mainly fesoterodine, tolterodine and solifenacin) of 1.13 (1.02–1.26) versus mirabegron. Although reporting greater risks for older patients, they did not examine gender differences. A New-Zealand case-time-control study reported a matched odds ratio (95% CI) for delirium with oxybutynin of 2.06 (1.07–3.96) and solifenacin of 0.89 (0.64–1.23) [37]. Although some studies on anticholinergics drugs in general report increased delirium risks, many have been inconsistent and underpowered, and limited by confounding by indication and residual confounding between patients [18].

### Mechanisms

Anticholinergic agents potentially exert a direct deleterious effect on the central nervous system as demonstrated by their cognitive effects [15]. Central cholinergic deficiency is a key hypothesised mechanism for delirium [4–7]. Trial evidence for acetylcholinesterase inhibitors reducing delirium risk is limited [4], but most studies are in people without cholinergic deficiency. However, a US observational study reported lower delirium rates in intensive care unit (ICU) admissions in people with dementia taking donepezil [38], and a trend to decreased postoperative delirium rates was detected in patients randomised to receive donepezil after elective total hip replacement [39]. Anticholinergic bladder medications can provoke urinary retention or constipation that increase UTI risk, a precipitating factor for delirium and hospital admissions [30]. However, our findings persisted after UTI adjustment.

Delirium rates remained elevated during treatment in men but not women. Gender differences have been noted in previous studies but not thoroughly explored [40–42]. Possible explanations include sex differences in the brain’s cholinergic system [43–45], greater comorbidity in men decreasing their ability to protect the brain from ‘chemical insults’ [4, 27] or depletion of susceptibles in very old women. Medication adherence is unlikely to be a key reason for the observed gender differences [46], as in these data, men were only slightly more likely to persist with long-term treatment than women [47].

### Implications for clinical practice

The results of our study will help inform the choice of OAB therapy for clinicians and patients in the shared decision-making process. Clinicians may wish to determine any history or other predisposing factors towards cognitive impairment or delirium. Alternative management strategies should be considered in these circumstances, as well as in frail older people. For instance, non-medical therapies including pelvic floor exercises, bladder training and other lifestyle modifications may prove less harmful for patients. If a bladder anticholinergic is required, prescribers should consider using lower doses in older people, with careful monitoring of efficacy and adverse effects to guide any dose adjustments [24]. Alternatively, patients and clinicians may wish to discuss using mirabegron, given that we did not identify an association between mirabegron and hospitalisation with delirium.

Healthcare professionals should obtain a detailed medication history, with specific enquiry regarding bladder and other anticholinergics, when assessing an older patient presenting with confusion or delirium [48]. Delirium may also arise from the interplay of several concurrent factors including infection, electrolyte abnormalities and compounded by the introduction of a bladder anticholinergic. Medication review, accompanied by withdrawal of any suspected predisposing medications or limitation on duration of use, should form an essential part of the management strategy, together with treatment of the physical causes.

Our findings also help inform future regulatory and prescribing guidance [22, 49, 50], particularly considering recent decisions to reject a UK licencing application for over-the-counter supply of oxybutynin through pharmacists [51]. Sufficient safeguards are needed to avoid drug-related adverse cognitive effects, especially in light of the evidence of only short-term, modest efficacy for bladder medications in relieving urgency symptoms and improving quality of life [52]. Our findings suggest the modest likelihood of benefit may not necessarily outweigh the risk of serious harm, thus providing supporting evidence for STOPP/START medication review guidelines [49].

## Conclusion

This SCCS links anticholinergic OAB prescriptions and increased hospital admission with delirium. Associations were broadly similar across drugs. Alternative OAB management should be prioritised in older adults, with anticholinergics used at the lowest effective dose and for limited duration. Clinical guidelines should explicitly weigh up potential benefits and harms of OAB in treatment pathways [53].

## Supplementary Material

aa-25-1856-File002_afaf308

## Data Availability

Data from the Clinical Practice Research Datalink cannot be shared by the authors but is available directly from CPRD. Full code lists corresponding to the exposures and each of the covariates we included are available on request. This study is based in part on data from the Clinical Practice Research Datalink obtained under licence from the UK Medicines and Healthcare products Regulatory Agency. The data is provided by patients and collected by the NHS as part of their care and support. The interpretation and conclusions contained in this study are those of the authors alone. Hospital Episode Statistics Admitted Patient Care data Copyright © (2021), was re-used with the permission of The Health & Social Care Information Centre. All rights reserved.
