# Supplementary material for: Overactive bladder medications and risk of emergency hospital admissions with delirium in adults without dementia: self-controlled case series
Source: Age Ageing. 2025 Oct 27;54(10):afaf308. doi: 10.1093/ageing/afaf308 (PMC12558043; doi:10.1093/ageing/afaf308)
Supplement: aa-25-1856-File002_afaf308 [file aa-25-1856-file002_afaf308.docx]

**Supplementary Material for Overactive bladder medications and risk of emergency hospital admissions with delirium in adults without dementia: self-controlled case series**

Contents

[Appendix 1. Self-controlled case series (SCCS) methods background 3](#_Toc207888721)

[Appendix 2. Defining prescription duration methods 4](#_Toc207888722)

[Appendix 3. Mirabegron SCCS analysis methods 5](#_Toc207888723)

[eFigure 1. Self-controlled case series study design examining anticholinergic OAB medication exposure and risk of delirium 6](#_Toc207888724)

[eFigure 2. Self-controlled case series study design examining mirabegron exposure and risk of delirium 6](#_Toc207888725)

[eFigure 3. Selection of patients with delirium admissions from the Clinical Practice Research Datalink (CPRD) Aurum 7](#_Toc207888726)

[eFigure 4. Distribution of time between admission with delirium and end of study period for patients initiating anticholinergic overactive bladder medications 8](#_Toc207888727)

[eFigure 5. Distribution of time between admission with delirium and end of study period for patients initiating mirabegron 9](#_Toc207888728)

[eTable 1. Primary diagnoses during hospital admission with delirium for patients prescribed either anticholinergic overactive bladder drugs or mirabegron 10](#_Toc207888729)

[eTable 2. 30-day incidence rates of emergency hospital admissions with delirium for patients initiating overactive bladder medications, by gender, year and age group 12](#_Toc207888730)

[eTable 3. Emergency hospital admissions between initiating anticholinergic overactive bladder medications and 31st December 2019 for patients without dementia with delirium recorded, by gender and year 13](#_Toc207888731)

[eTable 4. Incidence rate ratios for emergency hospital admission with delirium for patients initiating anticholinergic overactive bladder medications with five drug exposure periods, by gender 14](#_Toc207888732)

[eTable 5. Incidence rate ratios for emergency hospital admission with delirium for patients initiating anticholinergic overactive bladder medications, by gender, initial drug 15](#_Toc207888733)

[eTable 6. Incidence rate ratios for emergency hospital admission with delirium for patients initiating anticholinergic overactive bladder medications using standard self-control case series models, excluding those who died or transferred out within 90 days after delirium, by gender 17](#_Toc207888734)

[eTable 7. Incidence rate ratios for emergency hospital admission with delirium for patients initiating mirabegron using standard self-control case series models, excluding those who died or transferred out within 90 days after delirium 18](#_Toc207888735)

[eTable 8. Incidence rate ratios for emergency hospital admission with delirium for patients initiating anticholinergic overactive bladder medications with 60-day pre-exposure period, by gender 19](#_Toc207888736)

[eTable 9. Incidence rate ratios for emergency hospital admission with delirium for patients initiating anticholinergic overactive bladder medications without 30-day prescription end grace period, by gender 20](#_Toc207888737)

[eTable 10. Incidence rate ratios for emergency hospital admission with delirium for patients initiating anticholinergic overactive bladder medications excluding those diagnosed with dementia during the delirium admission, by gender 21](#_Toc207888738)

[eTable 11. Incidence rate ratios for emergency hospital admission with delirium for patients initiating mirabegron without a 30-day prescription end grace period or with 90-day pre-exposure period 22](#_Toc207888739)

[eTable 12. Incidence rate ratios for emergency hospital admission with delirium for patients initiating mirabegron excluding those with any history of OAB anticholinergic prescriptions 23](#_Toc207888740)

[References 24](#_Toc207888741)

## Appendix 1. Self-controlled case series (SCCS) methods background

The SCCS only include individuals who have experienced the outcome and makes comparisons within people who experience both the outcome and the exposure [1]. Conditional Poisson regression is used to estimated incidence rate ratios (IRRs) comparing event rates during exposed and unexposed periods.  This removes all fixed between-person confounding.

The standard SCCS assumes that occurrence of the outcome does not censor the observation period, for example, in the event of death. If there is evidence that the outcome censors the observation period by examining the distribution of remaining observation time after the event and performing sensitivity analyses, the SCCS extension for event-dependent observation periods can be applied [2]. The SCCS extension conditions on the age at censoring and weights cases by the density of the time from event to censoring.

## Appendix 2. Defining prescription duration methods

Prescription duration is sometimes missing in CPRD Aurum. Hence to estimate treatment episodes, we first prepared and cleaned the OAB prescription data [3]. Completely duplicated prescriptions were removed (2.7% of all OAB prescriptions). Prescriptions with quantity prescribed as implausible (<0.2%) or missing (<0.3%) values were replaced with the median quantity prescribed for each product in the complete prescription data. Prescriptions with dose frequency per day as implausible (<0.01%) or missing data (49%) were replaced with the prescription quantity/duration where duration was recorded. For the remaining prescriptions with missing dose frequency data (13%) due to duration being missing, we used the median dose frequency for each specific product in the complete data. Of these prescriptions with missing dose frequency data, the majority were for solifenacin (47%) and oxybutynin (32%). The imputations assumed the solifenacin was 1 tablet per day, and the oxybutynin was 1 per day for 13% of these prescriptions (products that were 5mg or 10mg) tablets and 2 per day for 87% of these prescriptions (products that were 2.5mg-5mg).

Prescription duration was estimated as quantity/dose frequency. We considered a new prescription represented a new treatment episode if it occurred >60 days after the previous prescription end date. The episode end was defined as the last prescription end date plus a 30-day grace period. These choices were informed by examining the Waiting Time Distribution [4], where 87% and 96% of patients returned for a subsequent prescription within 30 and 60 days, respectively, after their previous prescription ended [5].

## Appendix 3. Mirabegron SCCS analysis methods

We repeated the SCCS study design for patients initiating mirabegron. Due to fewer events, we expanded the study period to ≤12 months before initiating mirabegron (minimum 6 months), and ≤18 months after (eFigure 2). We excluded patients with an anticholinergic OAB prescription during the study period prior to mirabegron initiation or 30 days before. We adjusted for time-varying anticholinergic OAB prescriptions after mirabegron initiation. Due to fewer events, men and women were combined, an interaction with gender tested, and exposure periods categorised into risk windows: 1–30, 31–90, 91-180, and 181-545 days after prescription start date. We adjusted for age in ventiles of the delirium admission age distribution. As a recent delirium history may justify initiating an OAB patient on mirabegron rather than anticholinergics, we used two pre-exposure periods: 0 to -29, and -30 to -59 days.

## eFigure 1. Self-controlled case series study design examining anticholinergic OAB medication exposure and risk of delirium


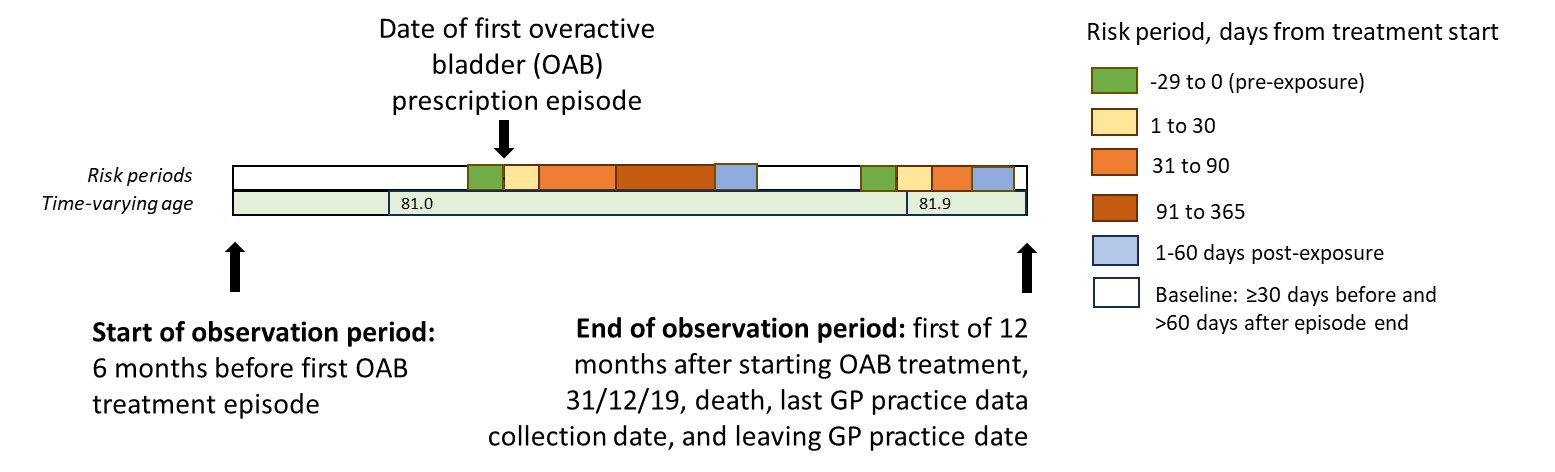


## eFigure 2. Self-controlled case series study design examining mirabegron exposure and risk of delirium


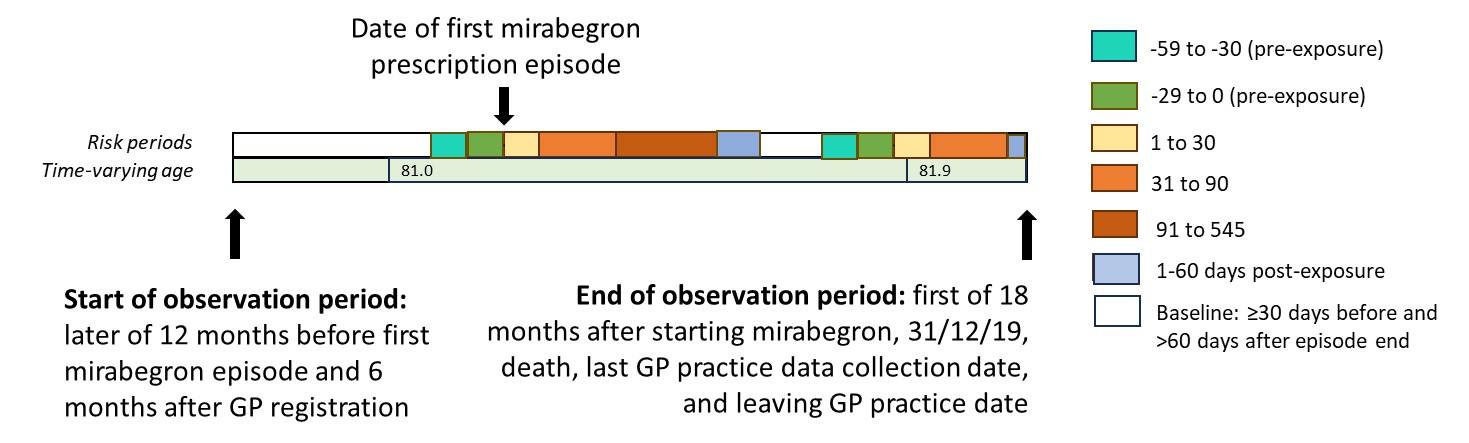


## eFigure 3. Selection of patients with delirium admissions from the Clinical Practice Research Datalink (CPRD) Aurum


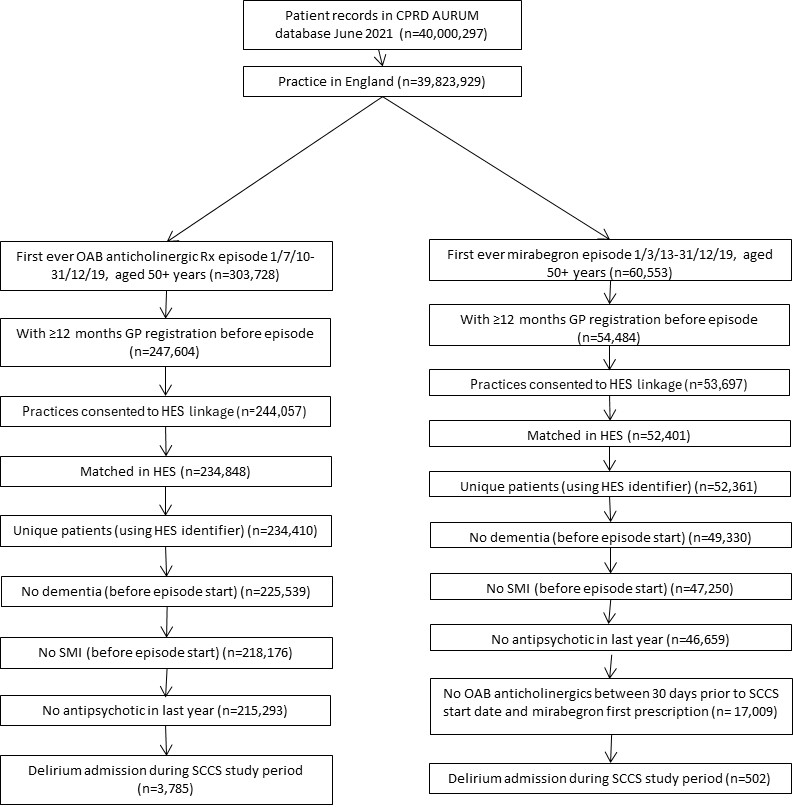


Abbreviations: HES, Hospital Episode Statistics; OAB, overactive bladder; SCCS Self-controlled case series; SMI, Severe mental illness

## eFigure 4. Distribution of time between admission with delirium and end of study period for patients initiating anticholinergic overactive bladder medications

## eFigure 5. Distribution of time between admission with delirium and end of study period for patients initiating mirabegron

## eTable 1. Primary diagnoses during hospital admission with delirium for patients prescribed either anticholinergic overactive bladder drugs or mirabegron

|  | **Initiating an OAB anticholinergic** | | | | **Initiating mirabegron** | | | |
| --- | --- | --- | --- | --- | --- | --- | --- | --- |
| **Primary diagnoses during hospital admission** | **Men (N=1831)** | | **Women (N=1954)** | | **Men (N=245)** | | **Women (N=257)** | |
| **with delirium*** | n | % | n | % | n | % | n | % |
| Delirium | 400 | 21.8% | 473 | 24.2% | 40 | 16.3% | 45 | 17.5% |
| Urinary tract/kidney infection | 363 | 19.8% | 403 | 20.6% | 39 | 15.9% | 42 | 16.3% |
| Pneumonia | 226 | 12.3% | 211 | 10.8% | 31 | 12.7% | 35 | 13.6% |
| Other lower respiratory tract infection | 67 | 3.7% | 64 | 3.3% | 8 | 3.3% | 16 | 6.2% |
| Other infection | 186 | 10.2% | 169 | 8.6% | 38 | 15.5% | 32 | 12.5% |
| Repeat falls/collapse/unsteadiness/dizziness | 119 | 6.5% | 162 | 8.3% | 19 | 7.8% | 19 | 7.4% |
| Fracture | 72 | 3.9% | 118 | 6.0% | 12 | 4.9% | 18 | 7.0% |
| Injury | 78 | 4.3% | 83 | 4.2% | 12 | 4.9% | 17 | 6.6% |
| Endocrine, nutritional and metabolic diseases | 85 | 4.6% | 98 | 5.0% | 12 | 4.9% | 14 | 5.4% |
| Diseases of the digestive system | 86 | 4.7% | 90 | 4.6% | 15 | 6.1% | 10 | 3.9% |
| Stroke/transient ischaemic attack | 47 | 2.6% | 69 | 3.5% | 11 | 4.5% | 6 | 2.3% |
| Cancer | 92 | 5.0% | 65 | 3.3% | 7 | 2.9% | 10 | 3.9% |
| Renal failure | 73 | 4.0% | 50 | 2.6% | 6 | 2.4% | <5 | <1.9% |
| Poisoning and certain other consequences of external causes | 52 | 2.8% | 16 | 0.8% | <5 | <2.0% | <5 | <1.9% |
| Other symptoms, signs and abnormal clinical and laboratory findings, not elsewhere classified | 173 | 9.4% | 173 | 8.9% | 12 | 4.9% | 23 | 8.9% |
| Other diseases of the circulatory system | 132 | 7.2% | 118 | 6.0% | 17 | 6.9% | 15 | 5.8% |
| Other diseases of the musculoskeletal system and connective tissue | 40 | 2.2% | 68 | 3.5% | 10 | 4.1% | 7 | 2.7% |
| Other diseases of the respiratory system | 52 | 2.8% | 47 | 2.4% | 14 | 5.7% | <5 | <1.9% |
| Other | 134 | 7.3% | 132 | 6.8% | 15 | 6.1% | 12 | 4.7% |

Abbreviations: OAB, Overactive bladder

* 1109 and 171 patients had 2 and 3-6 primary diagnosis codes listed for the emergency hospital admission

## eTable 2. 30-day incidence rates of emergency hospital admissions with delirium for patients initiating overactive bladder medications, by gender, year and age group

| Drug and group | Aged 50+ years | | | Aged 65+ years | | |
| --- | --- | --- | --- | --- | --- | --- |
|  | Delirium | Patient years | 30-day incidence | Delirium | Patient years | 30-day incidence |
|  | events |  | rate per 100 patients | events |  | rate per 100 patients |
| ***Anticholinergic*** | |  |  |  |  |  |
| Overall | 356 | 17599.4 | 0.17 | 335 | 11324.4 | 0.24 |
| Men | 181 | 7215.6 | 0.21 | 172 | 4963.6 | 0.28 |
| Women | 175 | 10383.8 | 0.14 | 163 | 6360.7 | 0.21 |
| Year |  |  |  |  |  |  |
| 2010 | 21 | 952.8 | 0.18 | 20 | 614.6 | 0.27 |
| 2011 | 39 | 2015.4 | 0.16 | 37 | 1297.2 | 0.23 |
| 2012 | 39 | 2105.9 | 0.15 | 38 | 1346.5 | 0.23 |
| 2013 | 36 | 2085.8 | 0.14 | 34 | 1348.8 | 0.21 |
| 2014 | 42 | 2058.7 | 0.17 | 36 | 1351.2 | 0.22 |
| 2015 | 45 | 1930.3 | 0.19 | 44 | 1271.2 | 0.28 |
| 2016 | 35 | 1853.7 | 0.16 | 32 | 1195.4 | 0.22 |
| 2017 | 26 | 1643.4 | 0.13 | 25 | 1041.7 | 0.20 |
| 2018 | 40 | 1518.1 | 0.22 | 40 | 954.4 | 0.34 |
| 2019 | 33 | 1435.3 | 0.19 | 29 | 903.3 | 0.26 |
| ***Mirabegron*** | |  |  |  |  |  |
| Overall | 27 | 1381.6 | 0.16 | 25 | 954.1 | 0.22 |

## eTable 3. Emergency hospital admissions between initiating anticholinergic overactive bladder medications and 31st December 2019 for patients without dementia with delirium recorded, by gender and year

|  |  | Emergency admissions |  |
| --- | --- | --- | --- |
| Group | Delirium  recorded |  | Percent with delirium |
| Overall | 14,250 | 190,333 | 7.5% |
| Men | 6,374 | 86,630 | 7.4% |
| Women | 7,876 | 103,703 | 7.6% |
| Year |  |  |  |
| 2010 | 1,076 | 16,354 | 6.6% |
| 2011 | 2,232 | 31,835 | 7.0% |
| 2012 | 2,255 | 30,968 | 7.3% |
| 2013 | 2,068 | 27,714 | 7.5% |
| 2014 | 1,959 | 25,466 | 7.7% |
| 2015 | 1,645 | 20,818 | 7.9% |
| 2016 | 1,289 | 16,495 | 7.8% |
| 2017 | 931 | 11,241 | 8.3% |
| 2018 | 590 | 6,880 | 8.6% |
| 2019 | 205 | 2,562 | 8.0% |

## eTable 4. Incidence rate ratios for emergency hospital admission with delirium for patients initiating anticholinergic overactive bladder medications with five drug exposure periods, by gender

|  | Men | | | |  | Women | | | |
| --- | --- | --- | --- | --- | --- | --- | --- | --- | --- |
|  | Delirium | Patient years | Incidence rate ratio (95% CI) | |  | Delirium | Patient years | Incidence rate ratio (95% CI) | |
| Time period | events |  | Adjusted for age | Adjusted for age & UTI |  | events |  | Adjusted for age | Adjusted for age & UTI |
| Baseline | 723 | 1113.2 | 1.00 | 1.00 |  | 829 | 1239.4 | 1.00 | 1.00 |
| Pre-exposure: 30 days before | 125 | 168.3 | 1.16 (0.96 to 1.41) | 0.99 (0.80 to 1.21) |  | 83 | 180.5 | 0.69 (0.55 to 0.87) | 0.60 (0.47 to 0.75) |
| Drug exposure: 1-30 days | 175 | 165.6 | 1.53 (1.29 to 1.82) | 1.33 (1.11 to 1.59) |  | 182 | 178.0 | 1.43 (1.22 to 1.69) | 1.26 (1.06 to 1.50) |
| Drug exposure: 31-90 days | 248 | 256.5 | 1.37 (1.17 to 1.60) | 1.25 (1.06 to 1.47) |  | 257 | 277.9 | 1.28 (1.10 to 1.49) | 1.25 (1.06 to 1.46) |
| Drug exposure: 91-180 days | 183 | 205.7 | 1.33 (1.10 to 1.61) | 1.24 (1.01 to 1.51) |  | 203 | 236.5 | 1.16 (0.97 to 1.39) | 1.12 (0.93 to 1.35) |
| Drug exposure: 181-270 days | 142 | 135.9 | 1.55 (1.23 to 1.94) | 1.40 (1.10 to 1.78) |  | 141 | 158.7 | 1.11 (0.89 to 1.38) | 1.02 (0.81 to 1.28) |
| Drug exposure: 271-365 days | 97 | 98.6 | 1.34 (1.02 to 1.77) | 1.17 (0.87 to 1.56) |  | 94 | 115.6 | 0.93 (0.71 to 1.21) | 0.84 (0.63 to 1.12) |
| Post-exposure: first 60 days | 138 | 173.1 | 0.97 (0.80 to 1.18) | 0.87 (0.71 to 1.07) |  | 165 | 191.4 | 1.07 (0.90 to 1.28) | 1.04 (0.87 to 1.25) |

Abbreviations: UTI, Urinary Tract Infection

## eTable 5. Incidence rate ratios for emergency hospital admission with delirium for patients initiating anticholinergic overactive bladder medications, by gender, initial drug

|  | Men | | |  | Women | | |
| --- | --- | --- | --- | --- | --- | --- | --- |
|  | Delirium | Incidence rate ratio (95% CI) | |  | Delirium | Incidence rate ratio (95% CI) | |
| Initial drug and time period | events | Age adjusted | Age & UTI adjusted |  | events | Age adjusted | Age & UTI adjusted |
| ***Oxybutynin*** |  |  |  |  |  |  |  |
| Baseline | 297 | 1.00 | 1.00 |  | 357 | 1.00 | 1.00 |
| Pre-exposure: 30 days before | 50 | 1.19 (0.87-1.61) | 0.98 (0.71-1.35) |  | 26 | 0.51 (0.34-0.77) | 0.42 (0.28-0.63) |
| Drug exposure: 1-30 days | 69 | 1.53 (1.17-2.01) | 1.34 (1.00-1.78) |  | 64 | 1.21 (0.92-1.60) | 1.05 (0.78-1.40) |
| Drug exposure: 31-90 days | 96 | 1.39 (1.08-1.77) | 1.27 (0.98-1.64) |  | 110 | 1.35 (1.08-1.70) | 1.33 (1.04-1.69) |
| Drug exposure: 91-365 days | 144 | 1.34 (1.02-1.75) | 1.19 (0.90-1.57) |  | 179 | 1.25 (0.98-1.59) | 1.17 (0.91-1.51) |
| Post-exposure: first 60 days | 59 | 0.96 (0.72-1.28) | 0.87 (0.64-1.19) |  | 71 | 1.05 (0.81-1.37) | 1.05 (0.80-1.39) |
| ***Solifenacin*** |  |  |  |  |  |  |  |
| Baseline | 240 | 1.00 | 1.00 |  | 260 | 1.00 | 1.00 |
| Pre-exposure: 30 days before | 45 | 1.19 (0.86-1.64) | 1.05 (0.75-1.48) |  | 37 | 0.94 (0.66-1.34) | 0.87 (0.61-1.25) |
| Drug exposure: 1-30 days | 70 | 1.74 (1.32-2.29) | 1.53 (1.14-2.05) |  | 71 | 1.71 (1.31-2.25) | 1.60 (1.20-2.14) |
| Drug exposure: 31-90 days | 90 | 1.33 (1.03-1.72) | 1.16 (0.88-1.53) |  | 86 | 1.25 (0.96-1.61) | 1.23 (0.93-1.61) |
| Drug exposure: 91-365 days | 163 | 1.33 (1.03-1.73) | 1.19 (0.90-1.57) |  | 165 | 1.09 (0.84-1.40) | 1.04 (0.80-1.35) |
| Post-exposure: first 60 days | 38 | 0.88 (0.62-1.26) | 0.76 (0.52-1.10) |  | 47 | 0.99 (0.72-1.37) | 0.94 (0.66-1.33) |
| ***Tolterodine*** |  |  |  |  |  |  |  |
| Baseline | 124 | 1.00 | 1.00 |  | 142 | 1.00 | 1.00 |
| Pre-exposure: 30 days before | 18 | 1.01 (0.61-1.67) | 0.77 (0.46-1.31) |  | 17 | 0.81 (0.49-1.36) | 0.67 (0.39-1.14) |
| Drug exposure: 1-30 days | 29 | 1.53 (1.01-2.33) | 1.26 (0.81-1.96) |  | 32 | 1.45 (0.98-2.16) | 1.27 (0.84-1.92) |
| Drug exposure: 31-90 days | 41 | 1.45 (0.99-2.11) | 1.35 (0.91-2.00) |  | 43 | 1.29 (0.90-1.85) | 1.23 (0.84-1.80) |
| Drug exposure: 91-365 days | 81 | 1.90 (1.29-2.80) | 1.76 (1.17-2.64) |  | 62 | 0.91 (0.62-1.32) | 0.88 (0.60-1.30) |
| Post-exposure: first 60 days | 26 | 1.01 (0.65-1.56) | 0.87 (0.55-1.39) |  | 38 | 1.44 (0.99-2.09) | 1.34 (0.90-1.99) |
| ***Other**** |  |  |  |  |  |  |  |
| Baseline | 62 | 1.00 | 1.00 |  | 70 | 1.00 | 1.00 |
| Pre-exposure: 30 days before | 12 | 1.29 (0.68-2.44) | 1.22 (0.63-2.38) |  | <5 | 0.31 (0.10-0.99) | 0.29 (0.09-0.93) |
| Drug exposure: 1-30 days | 7 | 0.71 (0.32-1.58) | 0.62 (0.27-1.43) |  | 15 | 1.44 (0.80-2.57) | 1.10 (0.59-2.04) |
| Drug exposure: 31-90 days | 21 | 1.32 (0.78-2.23) | 1.36 (0.79-2.36) |  | 18 | 1.15 (0.66-2.00) | 1.02 (0.56-1.85) |
| Drug exposure: 91-365 days | 34 | 0.99 (0.58-1.69) | 1.11 (0.64-1.93) |  | 32 | 0.96 (0.56-1.65) | 0.84 (0.48-1.47) |
| Post-exposure: first 60 days | 15 | 1.16 (0.64-2.09) | 1.16 (0.62-2.15) |  | 9 | 0.62 (0.30-1.28) | 0.63 (0.30-1.33) |

Abbreviations: UTI, Urinary Tract Infection

*Few patients initiated other anticholinergic OAB medications; trospium chloride (5%), fesoterodine (2%), darifenacin (1%), and propiverine (<1%).

## eTable 6. Incidence rate ratios for emergency hospital admission with delirium for patients initiating anticholinergic overactive bladder medications using standard self-control case series models, excluding those who died or transferred out within 90 days after delirium, by gender

|  | All patients | | | Excluding deaths within 90 days | | | Excluding death or transferred out within 90 days | | |
| --- | --- | --- | --- | --- | --- | --- | --- | --- | --- |
|  | Delirium | Incidence rate ratio (95% CI) | | Delirium | Incidence rate ratio (95% CI) | | Delirium | Incidence rate ratio (95% CI) | |
| Time period | events | Age adjusted | Age & UTI adjusted | events | Age adjusted | Age & UTI adjusted | events | Age adjusted | Age & UTI adjusted |
| ***Men*** |  |  |  |  |  |  |  |  |  |
| Baseline | 723 | 1.00 | 1.00 | 626 | 1.00 | 1.00 | 603 | 1.00 | 1.00 |
| Pre-exposure: 30 days before | 125 | 1.12 (0.92 to 1.35) | 0.94 (0.77 to 1.15) | 111 | 1.20 (0.98 to 1.47) | 1.01 (0.82 to 1.25) | 110 | 1.25 (1.02 to 1.53) | 1.05 (0.85 to 1.31) |
| Drug exposure: 1-30 days | 175 | 1.58 (1.33 to 1.87) | 1.36 (1.14 to 1.63) | 126 | 1.39 (1.14 to 1.69) | 1.20 (0.98 to 1.47) | 113 | 1.32 (1.07 to 1.62) | 1.14 (0.92 to 1.41) |
| Drug exposure: 31-90 days | 248 | 1.59 (1.33 to 1.89) | 1.51 (1.25 to 1.82) | 195 | 1.47 (1.21 to 1.79) | 1.39 (1.13 to 1.70) | 169 | 1.33 (1.08 to 1.63) | 1.28 (1.03 to 1.59) |
| Drug exposure: 91-365 days | 422 | 1.81 (1.52 to 2.16) | 1.71 (1.42 to 2.05) | 328 | 1.71 (1.42 to 2.07) | 1.63 (1.34 to 1.99) | 297 | 1.61 (1.32 to 1.95) | 1.55 (1.26 to 1.90) |
| Post-exposure: first 60 days | 138 | 1.13 (0.95 to 1.35) | 0.93 (0.77 to 1.11) | 99 | 0.98 (0.80 to 1.20) | 0.81 (0.65 to 1.00) | 92 | 0.98 (0.79 to 1.21) | 0.81 (0.65 to 1.01) |
| ***Women*** |  |  |  |  |  |  |  |  |  |
| Baseline | 829 | 1.00 | 1.00 | 753 | 1.00 | 1.00 | 714 | 1.00 | 1.00 |
| Pre-exposure: 30 days before | 83 | 0.67 (0.54 to 0.84) | 0.58 (0.46 to 0.73) | 77 | 0.72 (0.57 to 0.91) | 0.64 (0.50 to 0.81) | 75 | 0.74 (0.58 to 0.94) | 0.66 (0.51 to 0.84) |
| Drug exposure: 1-30 days | 182 | 1.47 (1.25 to 1.73) | 1.27 (1.07 to 1.52) | 143 | 1.34 (1.12 to 1.61) | 1.18 (0.97 to 1.43) | 133 | 1.34 (1.11 to 1.62) | 1.16 (0.95 to 1.42) |
| Drug exposure: 31-90 days | 257 | 1.26 (1.06 to 1.51) | 1.23 (1.02 to 1.48) | 213 | 1.15 (0.95 to 1.39) | 1.14 (0.93 to 1.39) | 199 | 1.13 (0.93 to 1.38) | 1.14 (0.92 to 1.40) |
| Drug exposure: 91-365 days | 438 | 1.24 (1.05 to 1.47) | 1.20 (1.01 to 1.43) | 371 | 1.13 (0.94 to 1.35) | 1.11 (0.92 to 1.33) | 335 | 1.10 (0.91 to 1.31) | 1.09 (0.90 to 1.31) |
| Post-exposure: first 60 days | 165 | 1.46 (1.25 to 1.69) | 1.35 (1.15 to 1.58) | 127 | 1.37 (1.16 to 1.62) | 1.26 (1.06 to 1.51) | 110 | 1.37 (1.15 to 1.63) | 1.28 (1.06 to 1.53) |

## eTable 7. Incidence rate ratios for emergency hospital admission with delirium for patients initiating mirabegron using standard self-control case series models, excluding those who died or transferred out within 90 days after delirium

|  | All patients | | | Excluding deaths within 90 days | | | Excluding death or transferred out within 90 days | | |
| --- | --- | --- | --- | --- | --- | --- | --- | --- | --- |
|  |  | Incidence rate ratio (95% CI) | |  | Incidence rate ratio (95% CI) | |  | Incidence rate ratio (95% CI) | |
| Time period | Delirium events | Adjusted for age and anticholinergic OAB exposure | Adjusted for age, anticholinergic OAB exposure & UTI | Delirium events | Adjusted for age and anticholinergic OAB exposure | Adjusted for age, anticholinergic OAB exposure & UTI | Delirium events | Adjusted for age and anticholinergic OAB exposure | Adjusted for age, anticholinergic OAB exposure & UTI |
| Baseline | 266 | 1.00 | 1.00 | 245 | 1.0 | 1.0 | 235 | 1.0 | 1.0 |
| Pre-exposure: 31-60 days before | 34 | 1.73 (1.21 to 2.49) | 1.54 (1.05 to 2.27) | 34 | 1.92 (1.34 to 2.77) | 1.73 (1.17 to 2.56) | 33 | 1.96 (1.35 to 2.83) | 1.76 (1.19 to 2.62) |
| Pre-exposure: 30 days before | 11 | 0.54 (0.30 to 1.00) | 0.44 (0.24 to 0.83) | 10 | 0.56 (0.29 to 1.05) | 0.42 (0.22 to 0.82) | 10 | 0.59 (0.31 to 1.11) | 0.44 (0.23 to 0.86) |
| Drug exposure: 1-30 days | 25 | 1.21 (0.80 to 1.84) | 1.15 (0.74 to 1.79) | 23 | 1.27 (0.82 to 1.96) | 1.19 (0.75 to 1.89) | 21 | 1.22 (0.78 to 1.92) | 1.15 (0.71 to 1.85) |
| Drug exposure: 31-90 days | 26 | 0.78 (0.52 to 1.18) | 0.77 (0.50 to 1.18) | 20 | 0.68 (0.43 to 1.09) | 0.68 (0.42 to 1.09) | 17 | 0.61 (0.37 to 1.01) | 0.61 (0.36 to 1.02) |
| Drug exposure: 91-545 days | 110 | 1.16 (0.85 to 1.57) | 0.94 (0.62 to 1.45) | 90 | 1.09 (0.79 to 1.51) | 0.90 (0.57 to 1.41) | 83 | 1.06 (0.76 to 1.49) | 0.90 (0.56 to 1.43) |
| Post-exposure: first 60 days | 30 | 1.21 (0.82 to 1.80) | 1.08 (0.71 to 1.64) | 23 | 1.14 (0.73 to 1.78) | 0.98 (0.61 to 1.58) | 22 | 1.20 (0.76 to 1.88) | 1.03 (0.64 to 1.67) |

## eTable 8. Incidence rate ratios for emergency hospital admission with delirium for patients initiating anticholinergic overactive bladder medications with 60-day pre-exposure period, by gender

|  | Men | | |  | Women | | |
| --- | --- | --- | --- | --- | --- | --- | --- |
|  | Delirium | Incidence rate ratio (95% CI) | |  | Delirium | Incidence rate ratio (95% CI) | |
| Time period | events | Age adjusted | Age & UTI adjusted |  | events | Age adjusted | Age & UTI adjusted |
| Baseline | 604 | 1.00 | 1.00 |  | 698 | 1.00 | 1.00 |
| Pre-exposure: 31-60 days before | 124 | 1.34 (1.10 to 1.63) | 1.26 (1.02 to 1.55) |  | 146 | 1.39 (1.16 to 1.67) | 1.20 (1.83 to 0.07) |
| Pre-exposure: 30 days before | 125 | 1.23 (1.01 to 1.50) | 1.04 (0.84 to 1.28) |  | 83 | 0.73 (0.57 to 0.92) | 0.61 (-4.00 to 0.00) |
| Drug exposure: 1-30 days | 175 | 1.62 (1.36 to 1.93) | 1.39 (1.15 to 1.67) |  | 182 | 1.51 (1.28 to 1.79) | 1.29 (2.79 to 0.01) |
| Drug exposure: 31-90 days | 246 | 1.44 (1.23 to 1.69) | 1.31 (1.10 to 1.55) |  | 252 | 1.33 (1.14 to 1.56) | 1.26 (2.82 to 0.00) |
| Drug exposure: 91-365 days | 421 | 1.46 (1.23 to 1.73) | 1.32 (1.10 to 1.59) |  | 436 | 1.15 (0.97 to 1.35) | 1.06 (0.66 to 0.51) |
| Post-exposure: first 60 days | 136 | 1.02 (0.84 to 1.24) | 0.92 (0.75 to 1.13) |  | 157 | 1.09 (0.91 to 1.31) | 1.04 (0.43 to 0.67) |

## eTable 9. Incidence rate ratios for emergency hospital admission with delirium for patients initiating anticholinergic overactive bladder medications without 30-day prescription end grace period, by gender

|  | Men | | |  | Women | | |
| --- | --- | --- | --- | --- | --- | --- | --- |
|  | Delirium | Incidence rate ratio (95% CI) | |  | Delirium | Incidence rate ratio (95% CI) | |
| Time period | events | Age adjusted | Age & UTI adjusted |  | events | Age adjusted | Age & UTI adjusted |
| Baseline | 795 | 1.00 | 1.00 |  | 896 | 1.00 | 1.00 |
| Pre-exposure: 30 days before | 125 | 1.16 (0.95 to 1.40) | 0.98 (0.80 to 1.21) |  | 83 | 0.69 (0.55 to 0.87) | 0.60 (0.47 to 0.76) |
| Drug exposure: 1-30 days | 171 | 1.54 (1.30 to 1.83) | 1.34 (1.11 to 1.60) |  | 177 | 1.44 (1.22 to 1.70) | 1.27 (1.06 to 1.51) |
| Drug exposure: 31-90 days | 181 | 1.45 (1.21 to 1.73) | 1.40 (1.16 to 1.69) |  | 173 | 1.20 (1.00 to 1.43) | 1.18 (0.98 to 1.42) |
| Drug exposure: 91-365 days | 388 | 1.48 (1.24 to 1.77) | 1.41 (1.17 to 1.69) |  | 394 | 1.07 (0.91 to 1.27) | 1.04 (0.87 to 1.24) |
| Post-exposure: first 60 days | 171 | 0.99 (0.83 to 1.18) | 0.82 (0.68 to 0.98) |  | 231 | 1.32 (1.13 to 1.53) | 1.24 (1.06 to 1.46) |

## eTable 10. Incidence rate ratios for emergency hospital admission with delirium for patients initiating anticholinergic overactive bladder medications excluding those diagnosed with dementia during the delirium admission, by gender

|  | Men | | | |  | Women | | | |
| --- | --- | --- | --- | --- | --- | --- | --- | --- | --- |
|  | Delirium | Patient years | Incidence rate ratio (95% CI) | |  | Delirium | Patient years | Incidence rate ratio (95% CI) | |
| Time period | events |  | Adjusted for age | Adjusted for age & UTI |  | events |  | Adjusted for age | Adjusted for age & UTI |
| Baseline | 680 | 1014.2 | 1.00 | 1.00 |  | 774 | 1118.4 | 1.00 | 1.00 |
| Pre-exposure: 30 days before | 125 | 153.9 | 1.21 (0.99 to 1.47) | 1.02 (0.83 to 1.25) |  | 82 | 163.9 | 0.71 (0.56 to 0.89) | 0.60 (0.47 to 0.76) |
| Drug exposure: 1-30 days | 163 | 151.3 | 1.51 (1.26 to 1.79) | 1.31 (1.09 to 1.58) |  | 165 | 161.6 | 1.36 (1.15 to 1.62) | 1.18 (0.98 to 1.42) |
| Drug exposure: 31-90 days | 223 | 234.4 | 1.32 (1.12 to 1.55) | 1.19 (1.00 to 1.41) |  | 235 | 252.1 | 1.25 (1.07 to 1.46) | 1.22 (1.03 to 1.43) |
| Drug exposure: 91-365 days | 364 | 401.5 | 1.33 (1.11 to 1.59) | 1.21 (1.01 to 1.46) |  | 377 | 469.2 | 1.00 (0.84 to 1.18) | 0.94 (0.79 to 1.13) |
| Post-exposure: first 60 days | 121 | 156.6 | 0.94 (0.77 to 1.16) | 0.84 (0.68 to 1.05) |  | 139 | 170.1 | 0.99 (0.82 to 1.20) | 0.95 (0.78 to 1.17) |

## eTable 11. Incidence rate ratios for emergency hospital admission with delirium for patients initiating mirabegron without a 30-day prescription end grace period or with 90-day pre-exposure period

|  | No 30-day grace period at end of treatment duration | | | Up to 90 days pre-exposure period | | |
| --- | --- | --- | --- | --- | --- | --- |
|  |  | Incidence rate ratio (95% CI) | |  | Incidence rate ratio (95% CI) | |
| Time period | Delirium events | Adjusted for age and anticholinergic OAB exposure | Adjusted for age, anticholinergic OAB exposure & UTI | Delirium events | Adjusted for age and anticholinergic OAB exposure | Adjusted for age, anticholinergic OAB exposure & UTI |
| Baseline | 280 | 1.00 | 1.00 | 280 | 1.00 | 1.00 |
| Pre-exposure: 61-90 days before | N/A | N/A | N/A | 34 | 1.27 (0.82 to 1.99) | 1.07 (0.67 to 1.69) |
| Pre-exposure: 31-60 days before | 34 | 1.79 (1.24 to 2.57) | 1.61 (1.09 to 2.38) | 11 | 1.84 (1.27 to 2.65) | 1.62 (1.09 to 2.40) |
| Pre-exposure: 30 days before | 11 | 0.53 (0.29 to 0.98) | 0.44 (0.23 to 0.83) | 25 | 0.55 (0.30 to 1.01) | 0.44 (0.23 to 0.83) |
| Drug exposure: 1-30 days | 25 | 1.15 (0.76 to 1.75) | 1.14 (0.73 to 1.77) | 12 | 1.17 (0.77 to 1.79) | 1.12 (0.72 to 1.74) |
| Drug exposure: 31-90 days | 19 | 0.67 (0.41 to 1.09) | 0.69 (0.42 to 1.14) | 20 | 0.73 (0.48 to 1.11) | 0.71 (0.46 to 1.09) |
| Drug exposure: 91-545 days | 100 | 0.80 (0.58 to 1.11) | 0.78 (0.56 to 1.09) | 87 | 0.88 (0.64 to 1.21) | 0.82 (0.59 to 1.14) |
| Post-exposure: first 60 days | 33 | 1.07 (0.73 to 1.55) | 0.95 (0.64 to 1.41) | 33 | 1.10 (0.74 to 1.63) | 0.97 (0.63 to 1.48) |

## eTable 12. Incidence rate ratios for emergency hospital admission with delirium for patients initiating mirabegron excluding those with any history of OAB anticholinergic prescriptions

|  |  | Incidence rate ratio (95% CI) | |
| --- | --- | --- | --- |
| Time period | Delirium events | Adjusted for age and anticholinergic OAB exposure | Adjusted for age, anticholinergic OAB exposure & UTI |
| Baseline | 232 | 1.00 | 1.00 |
| Pre-exposure: 31-60 days before | 9 | 2.17 (1.48 to 3.19) | 1.94 (1.28 to 2.93) |
| Pre-exposure: 30 days before | 19 | 0.57 (0.29 to 1.13) | 0.44 (0.22 to 0.89) |
| Drug exposure: 1-30 days | 11 | 1.17 (0.72 to 1.89) | 1.09 (0.65 to 1.81) |
| Drug exposure: 31-90 days | 17 | 0.87 (0.55 to 1.36) | 0.83 (0.52 to 1.33) |
| Drug exposure: 91-545 days | 63 | 0.96 (0.67 to 1.39) | 0.92 (0.63 to 1.34) |
| Post-exposure: first 60 days | 26 | 1.14 (0.71 to 1.83) | 0.97 (0.58 to 1.61) |

## References

**1**. Petersen I, Douglas I, Whitaker H. Self controlled case series methods: an alternative to standard epidemiological study designs. BMJ 2016; 354: i4515.

**2**. Farrington CP, Anaya-Izquierdo K, Whitaker HJ, Hocine MN, Douglas I, Smeeth L. Self-Controlled Case Series Analysis With Event-Dependent Observation Periods. J Am Stat Assoc 2011; 106: 417–426.

**3**. Pye SR, Sheppard T, Joseph RM *et al.* Assumptions made when preparing drug exposure data for analysis have an impact on results: An unreported step in pharmacoepidemiology studies. Pharmacoepidemiol Drug Saf 2018; 27: 781–788.

**4**. Pottegård A, Hallas J. Assigning exposure duration to single prescriptions by use of the waiting time distribution. Pharmacoepidemiol Drug Saf 2013; 22: 803–809.

**5**. Richardson K, Loke YK, Steel N *et al.* Time trends in the prescription of overactive bladder agents in England. PHARMACOEPIDEMIOLOGY AND DRUG SAFETY, WILEY 111 RIVER ST, HOBOKEN 07030-5774, NJ USA 2022, 180–180.
